# Chloroplast Genome Evolution of Hamamelidaceae at Subfamily Level

**DOI:** 10.1002/ece3.71141

**Published:** 2025-03-27

**Authors:** Yanlei Liu, Kangjia Liu, Wenpan Dong, Shunping Dong, Yiheng Wang, Chao Xu, Enze Li, Jiahui Sun

**Affiliations:** ^1^ School of Landscape and Ecological Engineering Hebei University of Engineering Handan China; ^2^ School of Ecology and Nature Conservation Beijing Forestry University Beijing China; ^3^ State Key Laboratory for Quality Ensurance and Sustainable Use of Dao‐Di Herbs，National Resource Center for Chinese Materia Medica Academy of Chinese Medical Sciences Beijing China; ^4^ State Key Laboratory of Plant Diversity and Specialty Crops Institute of Botany, Chinese Academy of Sciences Beijing China; ^5^ State Key Laboratory of Systematic and Evolutionary Botany Institute of Botany, Chinese Academy of Sciences Beijing China; ^6^ China National Botanical Garden Beijing China

**Keywords:** chloroplast genome, evolution, Hamamelidaceae, phylogeny

## Abstract

The Hamamelidaceae is significant for its contributions to construction, furniture making, and ornamental use, including 26 genera and 119 species. However, complete chloroplast genome sequences of Hamamelidaceae species have been reported less frequently. In this study, five species were newly sequenced, and seven others available complete chloroplast genomes were added to compare the chloroplast genome evolution in Hamamelidaceae at the subfamily level. The results indicated that the chloroplast genome size ranged from 158,116 to 159,941 bp, encoding 79 to 81 protein‐coding genes, four ribosomal RNA genes, and 30 to 31 transfer RNA genes. A robust phylogenetic tree of Hamamelidaceae was obtained using complete chloroplast genomes, supporting that all Hamamelidaceae species formed a monophyletic group and divided into four subfamilies. Exbucklandioideae was the first diverged group within Hamamelidaceae, followed by Mytilarioideae, Disanthoideae, and Hamamelidoideae, which formed a clade. Furthermore, three new potential DNA barcodes were provided: *trnH‐psbA*, *psbJ‐petA*, and *ycf1*. This study confirms that the complete chloroplast genome data provide a more accurate and confident resolution of the phylogenetic relationships within the Hamamelidaceae. These new genomic data not only enhance the understanding of genome evolution but also provide a better understanding of the phylogenetic relationships of Hamamelidaceae.

## Graphical abstract

The development of this study confirms that using the chloroplast whole genome can better and more confidently address the phylogenetic relationships of the Hamamelidaceae family. We also provide three reliable target regions for the identification of Hamamelidaceae species. These new genomic data are not only beneficial to access the genome evolution, but also provide information to elucidate the phylogenetic relationships of Hamamelidaceae.

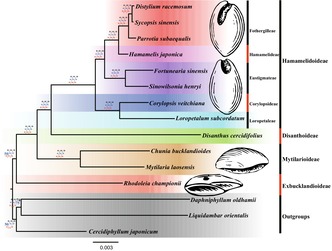

## 1 Introduction

There are approximately 119 species in 26 genera of Hamamelidaceae around the world, which are particularly distributed in eastern Asia, southern China (Zhang and Lu 1995). Most genera contain a small number of species; there are eight single‐species genera and only two genera of *Corylopsis* and *Distylium*, with more than 10 species. The fossil records indicated that Hamamelidaceae is an ancient group that was widely distributed across the Northern Hemisphere in the late Cretaceous (Zhang and Lu 1995). Hamamelidaceae plays a key role in understanding the origin and early evolution of core eudicots, making it essential for studying the phylogeny of this major group within angiosperms (Li et al. 2019). All plants in Hamamelidaceae are woody, and wood from *Exbucklandia*, *Chunia*, and *Mytilaria* is used for construction and furniture making. Additionally, species in *Hamamelis*, *Fortunearia*, and *Corylopsis* are used medicinally. Most genera also have ornamental value, with the most well‐known in this context being *Rhodoleia* and *Corylopsis* (Zhang et al. 2003). Many plants in Hamamelidaceae are protected. The IUCN Red List categorizes several species in Hamamelidaceae as follows: three critically endangered species (*Dicoryphe lanceolata*, *Embolanthera spicata*, and *Eustigma honbaense*), five endangered species (*Chunia bucklandioides*, *Dicoryphe buddleoides*, *Loropetalum subcordatum*, *Maingaya malayana*, *Matudaea trinervia*), and five vulnerable species (*Corylopsis coreana*, *Distyliopsis lanata*, *Distylium gracile*, *Hamamelis mexicana*, *Molinadendron sinaloense*). In China, *Parrotia subaequalis* is recognized as a first‐level protected plant, while *Chunia bucklandioides*, *Disanthus cercidifolius* subsp. *longipes*, and *Loropetalum subcordatum* have second‐level protection status.

The phylogeny of Hamamelidaceae has previously been reported using several chloroplast genes and/or nuclear gene markers; however, the phylogenetic relationships within Hamamelidaceae were not clearly resolved (Li et al. 1999; Wang et al. 2022; Xiang et al. 2019; Xie et al. 2010; Zhang et al. 2001). This may be because the markers used in previous studies were usually shorter and contained less information. Currently, phylogenetic trees have issues with low support values for some branches and cannot properly resolve the phylogeny of Hamamelidaceae. With the development of sequencing technology, particularly next generation sequencing (NGS), it has become easier to obtain complete chloroplast genome (CPG) sequences (Cui et al. 2024; Hu et al. 2021; Li et al. 2024; Liu et al. 2012; Slatko et al. 2018). More and more studies have used complete chloroplast genome data to resolve the phylogenetic relationships of the enigmatic groups (Chen et al. 2024; Sun et al. 2024; Yan et al. 2013). In Hamamelidaceae, Dong et al. (2021) used the complete chloroplast genome to conduct a phylogeny of *Distylium*. However, few studies focused on the evolution of chloroplast genomes in Hamamelidaceae at deep nodes.

According to recent phylogenies of some angiosperm groups, chloroplast genome sequences have had higher resolution for resolving the phylogeny (Alzahrani et al. 2020; Meng et al. 2024; Qian et al. 2021; Yang et al. 2013; Yaradua et al. 2019). With the continuous improvement of the APG (Angiosperm Phylogeny Group) system (Chase et al. 2016), the phylogenetic framework of high‐level classification of angiosperms has been mostly determined (such as at the order and family levels), but phylogenetic relationships below the family level have not been well resolved. Given that the phylogenies previously obtained using chloroplast and nuclear gene fragments have shown low branch support values, the phylogenetic relationships among genera of Hamamelidaceae are still not well resolved (Li et al. 1999; Xiang et al. 2019; Xie et al. 2010; Zhang and Lu 1995).

In this study, five species of *Corylopsis veitchiana*, *Disanthus cercidifolius*, *Hamamelis japonica*, *Rhodoleia championii*, and *Sinowilsonia henryi* were newly sequenced. The phylogenetic relationship of Hamamelidaceae at the subfamily level was inferred based on the complete chloroplast genome sequences. This approach will establish a foundation for species protection, while also paving the way for exploring the evolutionary history of Hamamelidaceae species.

## 2 Materials and Methods

### 2.1 Chloroplast Genome Sequencing and Assembly

The five Hamamelidaceae species materials were obtained from DNABank of the Institute of Botany, Chinese Academy of Sciences (Table 1). Genomic DNA was extracted using a modified CTAB method (Li et al. 2013). Using 1% agarose gel electrophoresis, the integrity and purity of DNA extractions were tested. Genomic DNA was fragmented into 350 bp segments using Covaris M220 to construct a sequencing library. The DNA library was sequenced using the Illumina Hiseq Platform X Ten, and each sample sequenced approximately four Gb (PE150) data. Trimmomatic v0.35 was used to filter out low‐quality fragments from the raw reads (Bolger et al. 2014). Get‐Organelle v1.6.2 was used to assemble the chloroplast genomes (Jin et al. 2020). In this manner, the complete chloroplast genome sequences of *Corylopsis veitchiana*, *Disanthus cercidifolius*, *Hamamelis japonica*, *Rhodoleia championii*, and *Sinowilsonia henryi* were obtained. Seven Hamamelidaceae chloroplast genome sequences were downloaded from GenBank (Table S1) for the following analyses.

**TABLE 1:** The basic chloroplast genome information of 12 Hamamelidaceae species.

Species	Sequence length (bp)				GC content (%)				Gene number			
	Whole genome	LSC	SSC	IR	Whole genome	LSC	SSC	IR	Total	CDs	rRNA	tRNA
*Chunia bucklandioides*	159,814	88,827	18,179	26,404	38.06	36.11	32.89	43.10	114	79	4	31
*Corylopsis veitchiana*	159,419	88,150	18,703	26,283	38.02	36.13	32.66	43.10	115	81	4	30
*Disanthus cercidifolius*	158,116	87,084	18,312	26,360	37.93	36.02	32.32	43.05	115	81	4	30
*Distylium racemosum*	159,107	87,863	18,782	26,231	38.01	36.18	32.46	43.07	113	79	4	30
*Fortunearia sinensis*	159,413	88,087	18,778	26,274	38.13	36.28	32.87	43.11	113	79	4	30
*Hamamelis japonica*	159,839	88,330	18,815	26,347	37.94	36.06	32.39	43.08	115	81	4	30
*Loropetalum subcordatum*	158,706	88,216	18,494	25,998	38.02	36.13	32.71	43.11	114	80	4	30
*Mytilaria laosensis*	159,941	89,016	18,127	26,399	37.91	35.90	32.76	43.07	113	79	4	30
*Parrotia subaequalis*	159,280	87,927	18,843	26,255	37.98	36.14	32.38	43.09	114	80	4	30
*Rhodoleia championii*	158,898	88,290	18,120	26,244	37.73	35.72	32.15	43.04	115	81	4	30
*Sinowilsonia henryi*	159,015	87,771	18,770	26,237	38.13	36.28	32.78	43.14	115	81	4	30
*Sycopsis sinensis*	159,043	87,805	18,778	26,230	38.02	36.19	32.46	43.06	113	79	4	30

### 2.2 Chloroplast Genome Annotation

The Perl script PGA was used to annotate the five obtained Hamamelidaceae chloroplast genomes (Qu et al. 2019). The related species of *Distylium racemosum* (MW248113) was used as the reference sequence. Geneious Prime 2021.1 was used to check and correct the annotation results (Kearse et al. 2012).

### 2.3 Characteristics Analysis of the Complete Chloroplast Genome

Through statistics and comparison of the basic characteristics of the complete chloroplast genome, it is possible to obtain an overview of the chloroplast genome and lay the foundation for subsequent research. A physical map of the Hamamelidaceae chloroplast genomes was performed using the OGDRAW function of MPI‐MP CHLOROBOX (Greiner et al. 2019). Geneious Prime 2021.1 was used to obtain basic information, such as chloroplast genome sequence length, GC content, length of the LSC (Large Single Copy region), SSC (Small Single Copy region), and IR (Inverted Repeat region) regions, and the numbers and types of genes.

### 2.4 Repeat Sequence Analysis

We used the online tool REPuter (https://bibiserv.cebitec.uni‐bielefeld.de/reputer) to count forward repeat sequences (Forward, F), inverted repeat sequences (Reverse, R), palindromic repeats (Palindromic, P) and complementary repeats (Complement, C). The minimum repeating unit was set to 30, and the Hamming distance was set to 3 (Kurtz et al. 2001). The Perl script Tandem Repeats Finder v4.10.0 was used to count the tandem repeat sequences. Match weight (Match), mismatch penalty score (Mismatch), insertion and deletion penalty score (Delta), match probability (PM), insertion probability (PI), minimum alignment score (Minscore), repeat fragment length, and the maximum value (MaxPeriod) were set to two, seven, seven, 80, 10, 50, and 500, respectively (Benson 1999). The Perl script MISA v2.1 was used to count the simple sequence repeats (SSR) (Beier et al. 2017). The numbers of mono‐, di‐, tri‐, tetra‐, penta‐, and hexanucleotide repeats were set to 10, six, five, four, three, and three, respectively. The minimum distance between two SSRs was set to 100 bp.

### 2.5 Comparative Analysis of Chloroplast Genome Differences

We used the Perl script to get mVISTA format from GenBank annotation (https://github.com/quxiaojian/Bioinformatic_Scripts/tree/master/get mVISTA format from GenBank annotation). The genome online analysis program mVISTA (http://genome.lbl.gov/vista/mvista/submit.shtml) was used to perform complete genome comparison and select the LAGAN mode; the sequence of *Mytilaria laosensis* was used as a reference for comparison (Frazer et al. 2004). Changes in chloroplast genome boundaries often contain evolutionary information. For example, contraction and expansion of IR regions often lead to significant changes in genome length. We used the R package IRscope to draw genes at boundary junctions and detect whether there was contraction and expansion of the IR region (Amiryousefi et al. 2018). The highly variable regions of the chloroplast genome of Hamamelidaceae were identified using nucleotide diversity (pi) values, which were calculated in DnaSP 6 (Rozas et al. 2017). Highly variable regions were identified through the sliding window method with window and step sizes set at 100 bp and 25 bp, respectively. The CDS Ka/Ks value was calculated using TBtools (Chen et al. 2020) and the RSCU analysis was conducted using Phylosuite (Zhang et al. 2020).

### 2.6 Phylogenetic Analysis

Three methods of Maximum parsimony (MP), Bayesian inference method (BI), and Maximum likelihood method (ML) were used to construct phylogenetic trees. The MP analyses were conducted using PAUP 4.0a169 (Swofford and Sullivan 2003). The ML method takes the topological structure, branch length, and other parts of the phylogenetic tree as parameters that need to be estimated. Based on the given data set and evolutionary model, the likelihood value is then maximized to estimate these parameters and obtain a phylogenetic tree. The BI method calculates the posterior probabilities of all possible branch types of evolutionary trees under a given data set and then merges the branches with the largest posterior probabilities to obtain a phylogenetic tree. In this study, PhyloSuite software was used to construct ML trees and BI trees. Three datasets were used for further analysis, including the complete chloroplast genome, the complete chloroplast genome with only one IR region, and all CDS regions. Three species were used as outgroups; the GenBank accession numbers used in this study were *Liquidambar orientalis* (MT079214), *Daphniphyllum oldhamii* (MH191390), and *Cercidiphyllum japonicum* (MN496059). All 15 chloroplast genomes were aligned using MAFFT v7.313 (Katoh and Standley 2013), and trimAl v1.2 was used to delete regions with unreliable alignments (Capella‐Gutiérrez et al. 2009).

ModelFinder was used to find suitable evolutionary models (Kalyaanamoorthy et al. 2017). IQ‐Tree v1.6.8 was used to construct ML trees (Nguyen et al. 2015), and bootstrap was set to standard mode with 500 repeated samplings. MrBayes v3.2.6 was used to construct the Bayesian tree (Ronquist et al. 2012). The model selected was GTR + F + I + G4 and was screened using ModelFinder. A total of 1,500,000 generations were run, and the remaining parameters were defaulted. The obtained phylogenetic tree was aesthetically modified on the online website ITOL (https://itol.embl.de), and the results were further processed with Adobe Illustrator 2021.

## 3 Results

### 3.1 Basic Characteristics of the Chloroplast Genome in Hamamelidaceae

**FIGURE 1:**
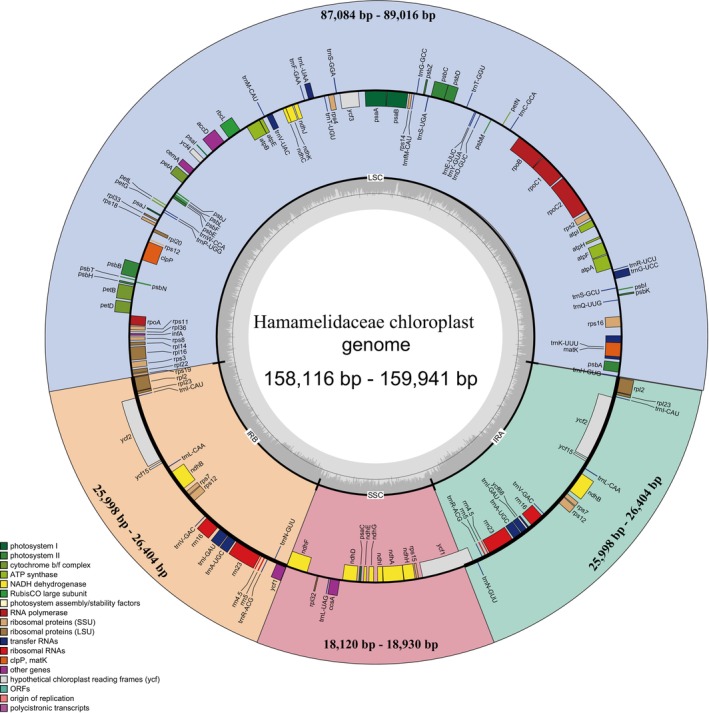
Physical chloroplast genome map of Hamamelidaceae.

The total length of Hamamelidaceae chloroplast genomes ranged from 158,116 to 159,941 bp. *Disanthus cercidifolius* was shortest, while *Mytilaria laosensis* was the longest. The chloroplast genome of Hamamelidaceae has the typical quadripartite structure, with LSC regions ranging from 87,084 to 89,016 bp, SSC regions ranging from 18,120 to 18,930 bp, and IR regions ranging from 25,998 to 26,404 bp. The GC content of the chloroplast genome ranged from 37.73% to 38.13%. In different regions, the GC content in the IR region was the highest, ranging from 43.04% to 43.14%, followed by the LSC region, which ranged from 35.72% to 36.28%. The lowest GC content was that of the SSC region, ranging from 32.15% to 32.89% (Figure 1).

The chloroplast genomes of Hamamelidaceae contain 113 to 115 unique genes, including 79 to 81 protein‐coding genes, four rRNA genes, and 30 to 31 tRNA genes. This difference in the number of protein‐coding genes is mainly due to the presence or absence of *ycf15* and *ycf68* genes. Other than *Chunia bucklandioides*, which had one more *trnP‐GGG* gene than other species, all other species had only 30 tRNA genes (Table 1). The chloroplast genome of Hamamelidaceae species showed a pair of reverse repeating IR regions, among which 18 genes had two copies in the IR region, including the protein‐coding genes: *ndhB*, *rpl2*, *rpl23*, *rps12*, *rps7*, *ycf15*, and *ycf2*; rRNA genes: *rrn16*, *rrn23, rrn4.5*, and *rrn5*; and tRNA genes: *trnA‐UGC*, *trnI‐CAU*, *trnI‐GAU*, *trnL‐CAA*, *trnN‐GUU*, *trnR‐ACG*, and *trnV‐GAC*.

The presence of introns was detected in 18 genes. Among them, *atpF*, *ndhA*, *ndhB*, *petB*, *petD*, *rpl2*, *rpl16*, *rpoC1*, *rps12*, *rps16*, *trnA‐UGC*, *trnG‐UCC*, *trnI‐GAU*, *trnK‐UUU*, *trnL‐UAA*, and *trnV‐UAC* genes had one intron, while *ycf3* and *clpP* had two introns. The protein‐coding gene *matK* is located in the intron region of the *trnK‐UUU* gene, and *ycf68* is located in the intron region of the *trnI‐GAU* gene. The *rps12* gene showed a trans‐splicing structure, with the 5′ end located in the LSC region and the 3′ end located in two IR regions (Table S2).

### 3.2 Repetitive Sequences and SSRs

**FIGURE 2:**
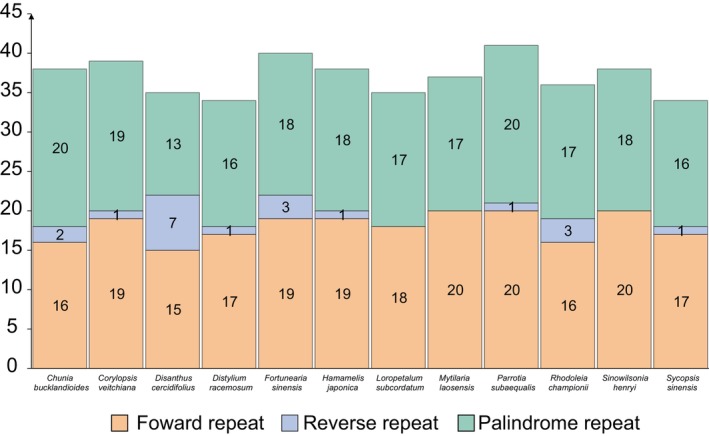
Frequency of tandem repeat sequences and dispersed repeat sequences in the 12 chloroplast genomes.

Twenty‐nine to 47 pairs of tandem repeat sequences were found in the chloroplast genome of Hamamelidaceae species, with the number for *Mytilaria laosensis* and *Rhodoleia championii* significantly higher than that of other species, at 44 and 47, respectively. The lowest number of tandem repeat sequences was 29 for *Sinowilsonia henryi*. The number of scattered repeat sequences ranged from 34 to 41 pairs. Among the repeat sequences scattered in different directions, there were more forward and palindromic repeat sequences. The number of reverse repeat sequences was relatively small, with most species ranging from zero to three pairs, while *Disanthus cercidifolius* had seven pairs of reverse repetitive sequences, significantly higher than that of other species. Additionally, no complementary repeat sequences were detected in the 12 Hamamelidaceae species (Figure 2).

**FIGURE 3:**
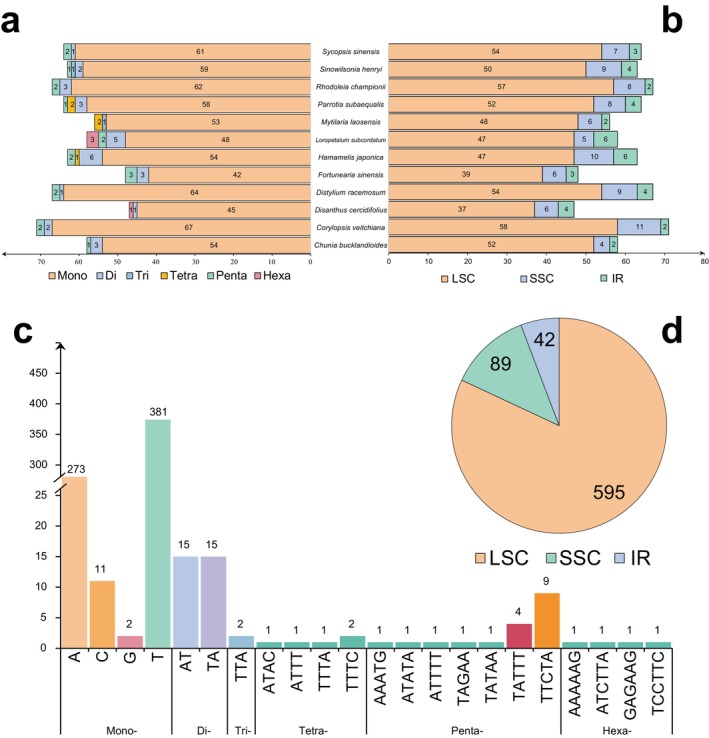
Distribution and frequency of the simple sequence repeat (SSR) with di‐ to hexa‐nucleotide motifs in the Hamamelidaceae chloroplast genome. (a) Number of different types of SSR in different species. (b) Number of SSR in different regions. (c) The distribution of different types of SSRs. (d) The distribution of SSRs in the LSC, SSC, and IR regions.

There were 47 to 71 SSR loci in the chloroplast genome of Hamamelidaceae species, with *Disanthus cercidifolius* showing the fewest SSR loci and *Corylopsis veitchiana* showing the most (Figure 3a,b). SSRs are mainly composed of single nucleotide repeats, comprising an average proportion of 91.87%, followed by dinucleotides and pentanucleotides, with average proportions of 4.13% and 2.48%, respectively. The main repeats in single nucleotides are A and T, which account for 52.48% and 37.60% of all SSRs, respectively. The type of dinucleotide is AT/TA, and the presence of dinucleotide repeats has been detected in all other species, except for *Mytilaria laosensis*. There are many types of repeat units in tetra‐, penta‐, and hexanucleotide repeats, but the number of each repeat unit is relatively small, typically amounting to one to two (Figure 3c). The SSR locus is mainly present in the LSC region, accounting for 74.60%–89.66%. Except for *Loropetalum subcordatum*, the number of SSR loci present in the IR region was the lowest, accounting for 2.82%–10.34% of the total SSR number (Figure 3d).

### 3.3 Chloroplast Genome Variations

The mVISTA results showed that similarity across the sequences, with most variations occurring in non‐coding regions, including the *psbZ‐trnG‐GCC*, *ycf3‐trnS‐GGA*, *petA‐psbJ*, and *ndhF‐rpl32* regions, where the sequence similarity was < 50%. The sequence similarity between tRNA and protein‐coding gene regions was high, with the similarity of most fragments higher than 90%. Among them, the most divergent genes were *ycf1*, *matK*, *rpoC2*, *accD*, and *ndhF*. The LSC region had the largest sequence difference, followed by the SSC region. The sequence differences between the two IR regions were smaller and more conservative (Figure S1).

The length of the IR region of different species ranged from 25,998 to 26,404 bp. The boundary genes of LSC‐IRb were *rps19* and *rpl2*, the boundary genes of IRb‐SSC were *ycf1* and *ndhF*, the boundary gene of SSC‐IRa was *ycf1*, and the boundary genes of IRa‐LSC were *rpl2* and *trnH*. The border of LSC‐IRb showed the smallest change. The *rps19* genes of seven species were one to 16 bp away from the border, while the borders of five species were located within the *rps19* gene. The length of the *rps19* genes in the IRb region of these five species was 2 bp. The boundaries of LSC‐IRa were all located between *rpl12* and *trnH*, and the distance from *trnH* to the boundary ranged from seven to 80 bp. The boundary difference between SSC‐IRb was the largest. Among genes located in the boundary areas, the length of the *ycf1* sequence varied greatly, ranging from 984 to 1136 bp. Most species had a segment of *ycf1* in the SSC region with a length of one to 38 bp, with only two exceptions; *Loropetalum subcordatum* was 2 bp from the SSC‐IRb boundary, and *Shaniodendron subaequale* was 45 bp from the boundary. The *ndhF* genes of most species were entirely located in the SSC region. Only *Corylopsis veitchiana* and *Mytilaria laosensis* had sections located in the LSC, which were 2 and 13 bp, respectively. Another *ycf1* gene spanned the SSC region and the IRa region, with a length of 4509 to 4596 bp in the SSC region and a length of 986 to 1107 bp in the IRa region (Figure S2).

**FIGURE 4:**
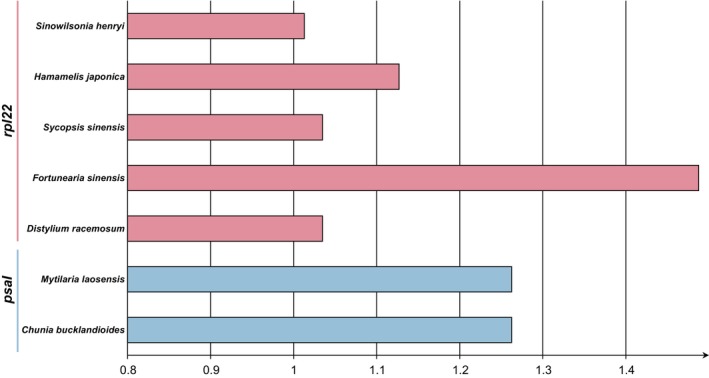
Genes under positive selection in the Hamamelidaceae.

This study found that species within the Hamamelidaceae exhibit similar trends in codon usage bias (Figure S3). In the calculation of Ka/Ks, positive selection was detected only in the *rps22* and *psaI* genes in some species. The rest of the genes showed purifying selection across all species (Figure 4).

**FIGURE 5:**
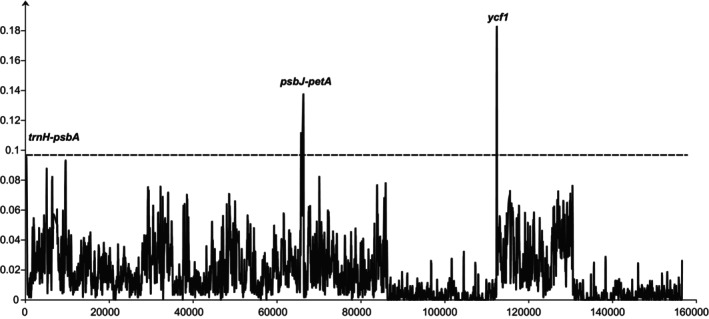
Variation of nucleotide polymorphisms with the position of the chloroplast genome.

Nucleotide diversity results were similar to the results of mVISTA. The Pi values of the two IR regions were relatively low; the nucleotide diversity of the LSC region and SSC region were relatively high. Three regions of *trnH‐psbA*, *psbJ‐PetA*, and *ycf1* exhibited the highest Pi values. *TrnH‐psbA* and *psbJ‐PetA* were located in the LSC region, and *ycf1* was located in the SSC region (Figure 5).

### 3.4 Phylogenetic Relationships of Hamamelidaceae

**FIGURE 6:**
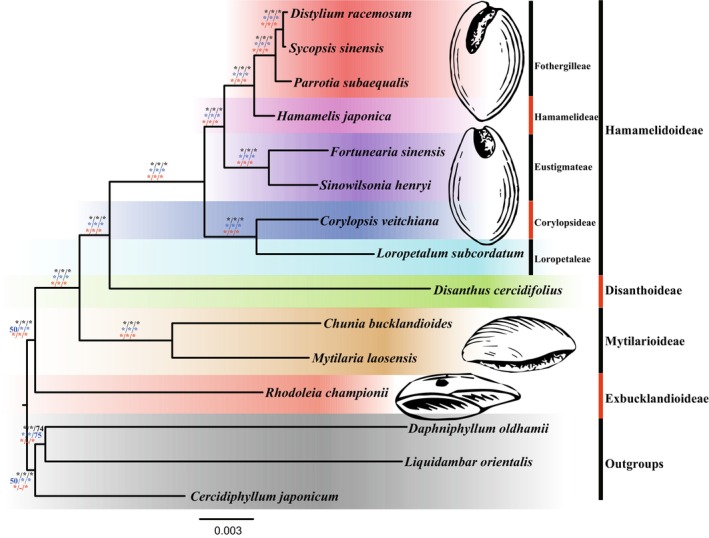
Phylogenetic relationships of Hamamelidaceae based on the chloroplast genome sequences. The support values are marked with the MP support values, BI posterior probabilities, and the ML support values. The results calculated from the complete chloroplast genome dataset are shown in black, those from the CDS region datasets are in blue, and those including only one IR region of the chloroplast genome dataset are in red. The sketch of seeds behind the phylogenetic tree represents the typical seed characteristics of each subfamily.

The three methods of MP, ML, and BI based on the three datasets showed consistent results of phylogenetic relationships of Hamamelidaceae. All the nodes had strong support values (Figure 6). All the trees supported Hamamelidaceae as a monophyletic group, and all four subfamilies of Hamamelidoideae, Disanthoideae, Mytilarioideae, and Exbucklandioideae formed monophyletic groups.

Among them, subfamily Exbucklandioideae was the first divergent group, followed by subfamily Mytilarioideae. Subfamily Disanthoideae and Hamamelidoideae were sister groups. The subfamily Hamamelidoideae was further divided into five tribes. Tribes Corylopsideae and Loropetaleae formed a clade, and Fothergilleae was sister to Hamamelideae.

## 4 Discussion

### 4.1 The Chloroplast Genome Evolution of Hamamelidaceae

Through comprehensive analysis of Hamamelidaceae chloroplast genomes, it was observed that the length of the chloroplast genomes varied by 1825 bp. All genomes exhibited a quadripartite structure, which was consistent with the most common structure in angiosperms (Liu et al. 2024; Pombert et al. 2006; Wang et al. 2025, 2023). The gene types, numbers, and orders were conserved, with minimal differences in GC content. SSR analysis revealed that single nucleotide repeats were predominant, constituting over 90% of total SSRs, while other SSR types were less prevalent (Dong et al. 2021). This discrepancy may be attributed to the set detection thresholds, which may exclude other repeat types.

The IR regions demonstrated less variation compared to the LSC and SSC regions, with the LSC region exhibiting numerous sites with < 50% similarity, which is also the same as the most chloroplast genomes in angiosperms (Dong et al. 2021; Liu et al. 2024; Pombert et al. 2006; Wang et al. 2025; Zhao et al. 2018). Non‐coding regions displayed more sequence variation than coding regions, likely due to the significant impact of coding region mutations on gene function and protein expression, imposing higher selective pressure and resulting in relatively smaller differences (Zhang et al. 2021). The IR region of the chloroplast genome is generally conserved, but it can also undergo expansion and contraction in the boundary with other regions (LSC and SSC), influencing genome length (Zhao et al. 2018). In Hamamelidaceae, there are fewer variations in the boundary between the LSC region and IR regions, while more pronounced changes were noted between the SSC and IR regions. The *ycf1* gene sequence lengths at these boundaries varied significantly, whereas the *ndhF* gene sequence lengths showed minor differences, with the *ndhF* gene located 13 bp within the IRb region to 52 bp from the LSC‐IRb boundary, indicating some degree of IR region expansion and contraction.

### 4.2 Phylogenetic Relationships of Hamamelidaceae at Subfamily Level

The resulting MP, ML, and BI trees exhibited robust branch support, with consistent topology and branch lengths. Previous phylogenetic studies utilized nuclear ITS fragments and/or several chloroplast fragments, resulting in low support for some branches due to lower information in those genetic markers (Li et al. 1999; Xiang et al. 2019; Xie et al. 2010). For example, Xiang et al. (2019) constructed a phylogenetic tree using both MP and ML methods but encountered low support values for certain subfamilies, likely due to incomplete sequence data. In this study, the complete chloroplast genomes provided more comprehensive phylogenetic information and yielded robust phylogenetic relationships of Hamamelidaceae at deep nodes (Figure 6), demonstrating the superior reliability of complete chloroplast genomes for phylogenetic analysis. Unfortunately, genetic data on Hamamelidaceae are still limited at present. This study offers a valuable reference for future phylogenetic research and species identification within this family.

### 4.3 Potential Highly Variable DNA Barcodes

Numerous studies have highlighted the limitations of universal DNA barcodes in terms of divergence and discriminatory power (Dong et al. 2014, 2012; Li et al. 2011; Hollingsworth et al. 2009; Kress 2017; Kress and Erickson 2007). Hamamelidaceae species are significant both as research subjects and economic resources, but the paucity of genomic data poses challenges for taxonomy, genetics, identification, and conservation efforts. More and more results show the complete chloroplast genome sequences contain more variable information and can be widely used as a DNA barcode for species identification (Liu, Guo, et al. 2025; Liu, Chen, et al. 2025).

Mutation events in the chloroplast genome are not uniformly distributed but are concentrated in specific “hotspot” regions (Dong et al. 2012; Hebert et al. 2003; Kress 2017; Kress and Erickson 2007). Comparative analysis of chloroplast genomes is an effective strategy for identifying these hypervariable regions, which can serve as taxon‐specific DNA barcodes. This study identified three hypervariable regions, including *trnH‐psbA*, *psbJ‐petA*, and *ycf1*. Previous research has advocated for *trnH‐psbA* and *ycf1* as complementary barcodes to the universal barcode for seed plants (Dong et al. 2015; Kress and Erickson 2007; Pang et al. 2012). However, *psbJ‐petA* has not been extensively used in plant phylogeny and DNA barcoding, being a specific barcode for Hamamelidaceae species. These regions have the potential for the accurate identification of Hamamelidaceae species, though further verification with a broader range of species within the family is still needed.

## 5 Conclusions

In this study, a comprehensive analysis of the Hamamelidaceae chloroplast genome was conducted. The Hamamelidaceae chloroplast genome exhibited a typical quadripartite structure and was relatively conserved. Among the three major regions, the IR region exhibited the smallest differences, while the LSC region showed the largest. Nucleotide diversity results provide three novel candidate DNA barcodes for the identification of Hamamelidaceae species. Phylogenetic results based on the chloroplast genome sequences indicated that Hamamelidaceae was monophyletic, with each of its four subfamilies forming distinct branches. Hamamelidoideae was further divided into five tribes. The phylogenetic tree demonstrated higher branch support, underscoring the utility of chloroplast genome sequences in elucidating the phylogenetic relationships of Hamamelidaceae.

## Author Contributions

**Yanlei Liu:** conceptualization (equal), formal analysis (equal), funding acquisition (equal), investigation (equal), resources (equal), supervision (equal), validation (equal), visualization (equal), writing – original draft (equal), writing – review and editing (equal). **Kangjia Liu:** data curation (equal), investigation (equal), resources (equal). **Wenpan Dong:** resources (equal), supervision (equal), validation (equal), visualization (equal), writing – original draft (equal), writing – review and editing (equal). **Shunping Dong:** formal analysis (equal), investigation (equal), methodology (equal). **Yiheng Wang:** resources (equal), validation (equal), visualization (equal). **Chao Xu:** resources (equal), visualization (equal). **Enze Li:** conceptualization (equal), writing – original draft (equal), writing – review and editing (equal). **Jiahui Sun:** conceptualization (equal), resources (equal), supervision (equal), writing – original draft (equal), writing – review and editing (equal).

## Ethics Statement

Experimental research and field studies on plants (either cultivated or wild), including the collection of plant material, comply with relevant institutional, national, and international guidelines and legislation.

## Consent

The authors have nothing to report.

## Conflicts of Interest

The authors declare no conflicts of interest.

## Supporting information


**Figure S1.** Results of mVISTA analysis showed differences in the sequences among Hamamelidaceae species.

**Figure S2.** Comparison of the borders among the 12 Hamamelidaceae chloroplast genomes.

**Figure S3.** Codon usage in Hamamelidaceae chloroplast genome.

**Table S1.** The basic information of 12 Hamamelidaceae species.

**Table S2.** Gene composition of Hamamelidaceae chloroplast genome.

## Data Availability

All five complete chloroplast genome data have been submitted to GenBank; the accession numbers of each genome are OR726642, OR726643, OR726644, OR726645, and OR726646.
