# Supplementary figures and images for: Chloroplast Genome Evolution of Hamamelidaceae at Subfamily Level
Source: Ecol Evol. 2025 Mar 27;15(4):e71141. doi: 10.1002/ece3.71141 (PMC11949566; doi:10.1002/ece3.71141)

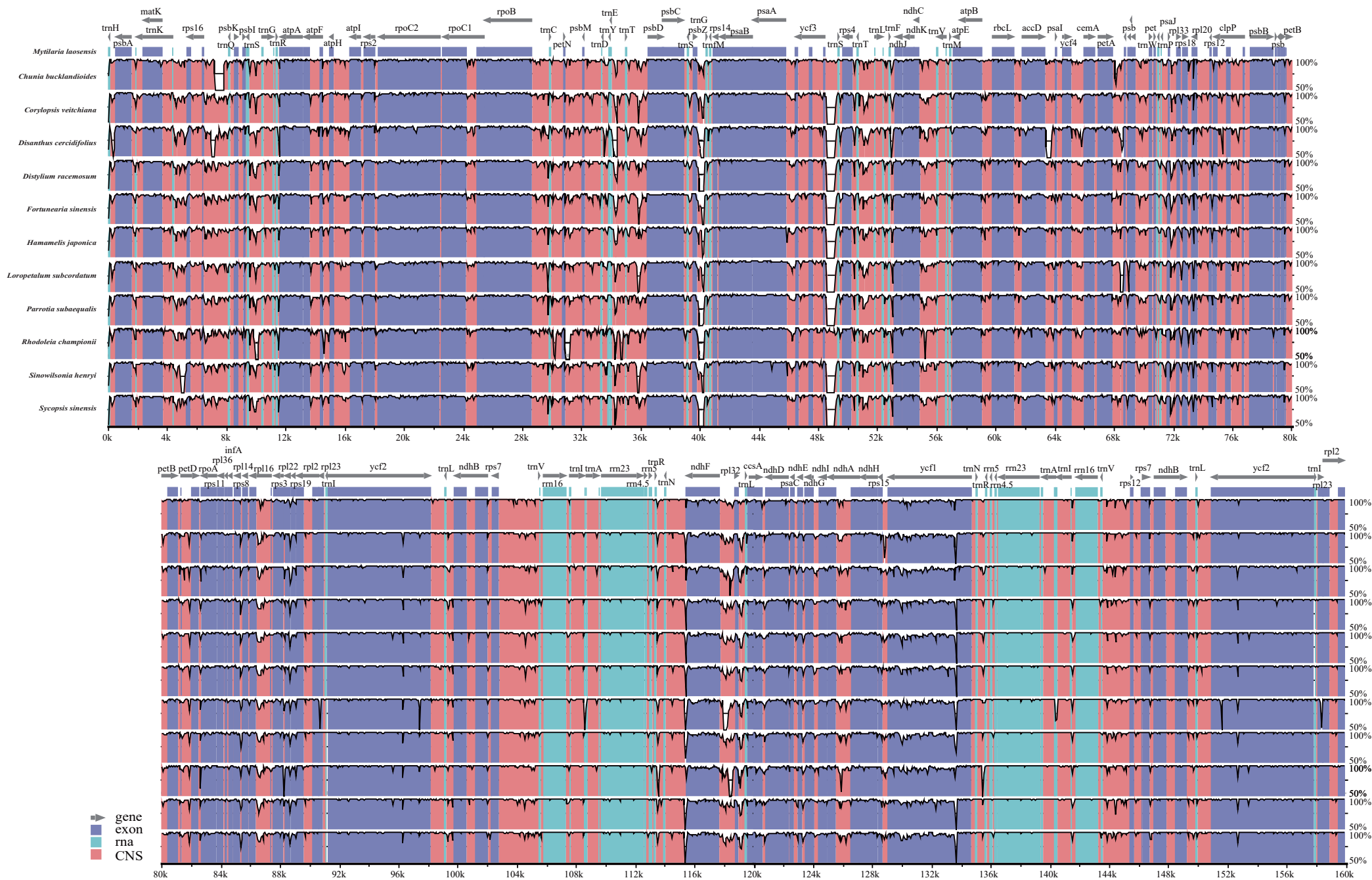

Supplement: Supplementary file 1 — Figure S1. Results of mVISTA analysis showed differences in the sequences among Hamamelidaceae species. [file ECE3-15-e71141-s003.pdf]

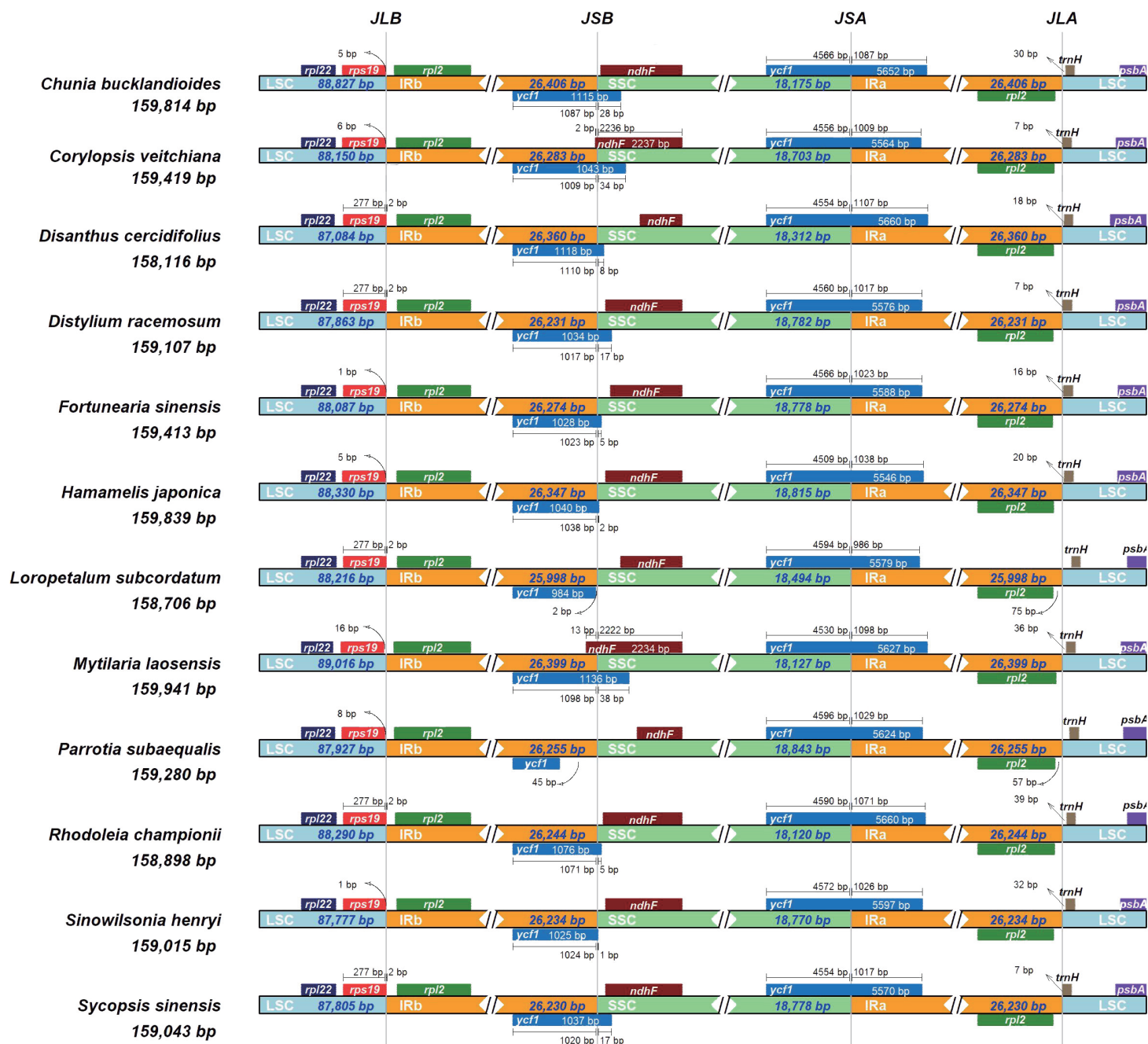

Supplement: Supplementary file 2 — Figure S2. Comparison of the borders among the 12 Hamamelidaceae chloroplast genomes. [file ECE3-15-e71141-s002.pdf]

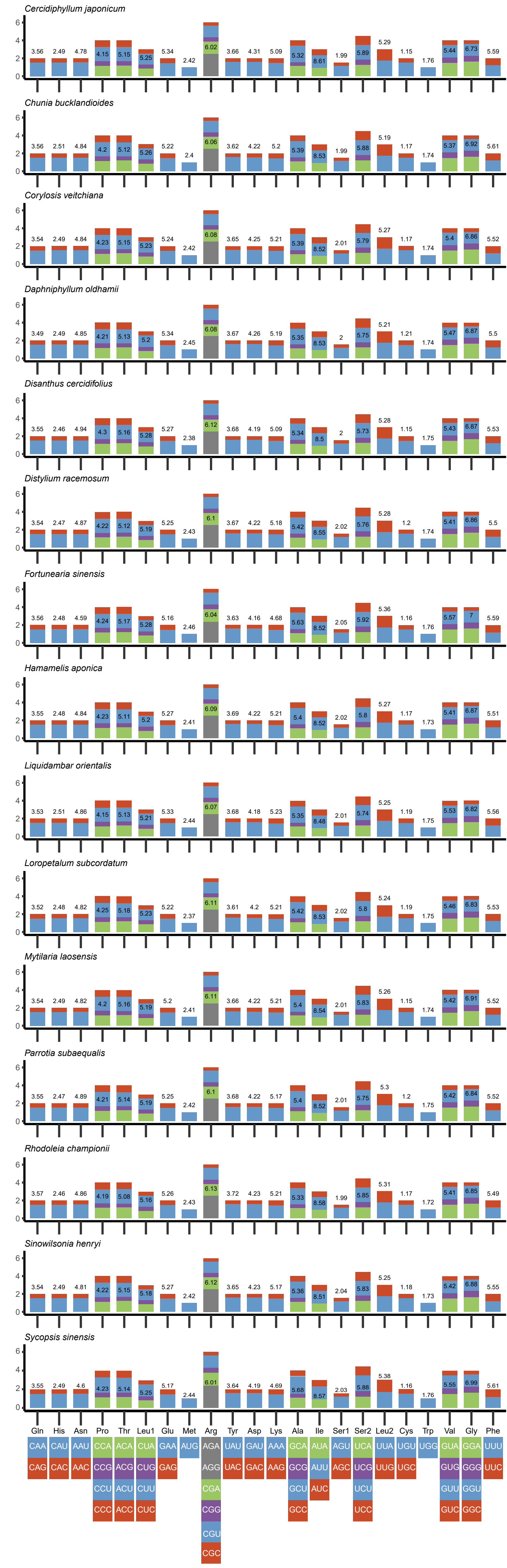

Supplement: Supplementary file 3 — Figure S3. Codon usage in Hamamelidaceae chloroplast genome. [file ECE3-15-e71141-s001.pdf]
