# Supplementary material for: Chloroplast Genome Evolution of Hamamelidaceae at Subfamily Level
Source: Ecol Evol. 2025 Mar 27;15(4):e71141. doi: 10.1002/ece3.71141 (PMC11949566; doi:10.1002/ece3.71141)
Supplement: Supplementary file 4 — Table S1. The basic information of 12 Hamamelidaceae species. [file ECE3-15-e71141-s005.docx]

**Table S1 The basic information of 12 Hamamelidaceae species.**

| **Species** | **Original number** | **Source** | **Specimen number** | **GenBank number** | **Appraiser** | **Location** |
| --- | --- | --- | --- | --- | --- | --- |
|  |  |  |  |  |  |  |
| ***Chunia bucklandioides*** | Public data |  |  | NC041163 |  |  |
| ***Corylopsis veitchiana*** | HUE000001 | DNABank of IBCAS | 01801072 | OR726642 | Qixin Liu | Anhui, China |
| ***Disanthus cercidifolius*** | HUE000002 | DNABank of IBCAS | 01841878 | OR726643 | Zhiyun Zhang | Jiangxi, China |
| ***Distylium racemosum*** | Public data |  |  | MW248113 |  |  |
| ***Fortunearia sinensis*** | Public data |  |  | MN496061 |  |  |
| ***Hamamelis japonica*** | HUE000003 | DNABank of IBCAS | 01149794 | OR726644 | Miyoshi Furuse | Japan |
| ***Loropetalum subcordatum*** | Public data |  |  | NC037694 |  |  |
| ***Mytilaria laosensis*** | Public data |  |  | NC048997 |  |  |
| ***Parrotia subaequalis*** | Public data |  |  | NC037243 |  |  |
| ***Rhodoleia championii*** | HUE000004 | DNABank of IBCAS | 00803144 | OR726645 | S.Y.Hu | Hongkong, China |
| ***Sinowilsonia henryi*** | HUE000005 | DNABank of IBCAS | 02062386 | OR726646 | Zhiyun Zhang | Beijing, China |
| ***Sycopsis sinensis*** | Public data |  |  | MN496080 |  |  |

DNABank of IBCAS = DNABank of Institute of Botany, Chinese Academy of Sciences
