# Supplementary material for: Chloroplast Genome Evolution of Hamamelidaceae at Subfamily Level
Source: Ecol Evol. 2025 Mar 27;15(4):e71141. doi: 10.1002/ece3.71141 (PMC11949566; doi:10.1002/ece3.71141)
Supplement: Supplementary file 5 — Table S2. Gene composition of Hamamelidaceae chloroplast genome. [file ECE3-15-e71141-s004.docx]

| **Table S2 Gene composition of chloroplast genome of 12 Hamamelidaceae species.** | | |
| --- | --- | --- |
| Gene classification | Gene Group | Gene name |
| Photosynthetic genes | Photosystem I gene | *psaA,psaB,psaC,psaI,psaJ* |
|  | Photosystem II gene | *psbA,psbB,psbC,psbD,psbE,psbF,psbH,psbI,psbJ,psbK,psbL,psbM,psbN,psbT,psbZ* |
|  | NADH dehydrogenase gene | *ndhA*,ndhB*(2),ndhC,ndhD,ndhE,ndhF,ndhG,ndhH,ndhI,ndhJ,ndhK* |
|  | Cytochrome complex gene | *petA,petB*,petD*,petG,petL,petN* |
|  | ATP synthase gene | *atpA,atpB,atpE,atpF*,atpH,atpI* |
|  | Rubisco large subunit gene | *rbcL* |
| Transcription translation genes | Large ribosome subunit gene | *rpl14,rpl16*,rpl2*(2),rpl20,rpl22,rpl23(2),rpl32,rpl33,rpl36* |
|  | Small ribosome subunit gene | *rps11,rps12,rps12*(2),rps14,rps15,rps16*,rps18,rps19,rps2,rps3,rps4,rps7(2),rps8* |
|  | RNA polymerase gene | *rpoA, rpoB,rpoC1*,rpoC2* |
|  | Ribosome RNA gene | *rrn16(2),rrn23(2),rrn4.5(2),rrn5(2)* |
|  | Transport RNA gene | *trnA-UGC*(2),trnC-GCA,trnD-GUC,trnE-UUC,trnF-GAA,trnG-GCC,trnG-UCC*,trnH-GUG,trnI-CAU(2),trnI-GAU*(2),trnK-UUU*,trnL-CAA(2),trnL-UAA*,trnL-UAG,trnM-CAU,trnN-GUU(2),trnP-UGG,trnQ-UUG,trnR-ACG(2),trnR-UCU,trnS-GCU,trnS-GGA,trnS-UGA,trnT-GGU,trnT-UGU,trnV-GAC(2),trnV-UAC*,trnW-CCA,trnY-GUA,trnfM-CAU* |
| Biosynthetic genes | Maturase K gene | *matK* |
|  | Protease gene | *clpP*** |
|  | Membrane protein gene | *cemA* |
|  | Acetyl-CoA carboxylase gene | *accD* |
|  | C-type cytochrome synthesis gene | *ccsA* |
|  | transcription initiation factor gene | *infA* |
| unknown genes | Conserved open reading frame | *ycf1,ycf15(2),ycf2(2),ycf3**,ycf4,ycf68* |
| Note: * indicates that the gene has one intron, * * indicates that the gene has two introns, and (2) indicates that the gene has two copies | | |
